# Augmenting melodic intonation therapy with non-invasive brain stimulation to treat impaired left-hemisphere function: two case studies

**DOI:** 10.3389/fpsyg.2014.00037

**Published:** 2014-02-04

**Authors:** Shahd Al-Janabi, Lyndsey A. Nickels, Paul F. Sowman, Hana Burianová, Dawn L. Merrett, William F. Thompson

**Affiliations:** ^1^ARC Centre of Excellence in Cognition and its Disorders, Macquarie UniversitySydney, NSW, Australia; ^2^Department of Cognitive Science, Macquarie UniversitySydney, NSW, Australia; ^3^NHMRC Centre of Clinical Research Excellence in Aphasia Rehabilitation, Macquarie UniversitySydney, NSW, Australia; ^4^Centre for Advanced Imaging, The University of QueenslandBrisbane, QLD, Australia; ^5^Melbourne School of Psychological Sciences, The University of MelbourneMelbourne, VIC, Australia; ^6^Department of Psychology, Macquarie UniversitySydney, NSW, Australia

**Keywords:** aphasia, stroke, fMRI, rTMS, rehabilitation

## Abstract

The purpose of this study was to investigate whether or not the right hemisphere can be engaged using Melodic Intonation Therapy (MIT) and excitatory repetitive transcranial magnetic stimulation (rTMS) to improve language function in people with aphasia. The two participants in this study (GOE and AMC) have chronic non-fluent aphasia. A functional Magnetic Resonance Imaging (fMRI) task was used to localize the right Broca's homolog area in the inferior frontal gyrus for rTMS coil placement. The treatment protocol included an rTMS phase, which consisted of 3 treatment sessions that used an excitatory stimulation method known as intermittent theta burst stimulation, and a sham-rTMS phase, which consisted of 3 treatment sessions that used a sham coil. Each treatment session was followed by 40 min of MIT. A linguistic battery was administered after each session. Our findings show that one participant, GOE, improved in verbal fluency and the repetition of phrases when treated with MIT in combination with TMS. However, AMC showed no evidence of behavioral benefit from this brief treatment trial. Post-treatment neural activity changes were observed for both participants in the left Broca's area and right Broca's homolog. These case studies indicate that a combination of MIT and rTMS applied to the right Broca's homolog has the potential to improve speech and language outcomes for at least some people with post-stroke aphasia.

## Introduction

The ability to articulate ideas and comprehend speech, sign language, and writing is vital to participating in human society. This important skill can be disrupted by damage to the language areas that are usually localized in the left hemisphere of the brain. One-third of people who have had left hemispheric stroke suffer from aphasia; that is, they have difficulty in producing and/or comprehending language (Aftonomos et al., 1999). Stroke patients who experience aphasia commonly require extensive therapy to improve their ability to communicate. The traditional therapy approach to post-stroke language recovery is focused on teaching compensatory strategies or on repetitive training of lost function. These speech therapies have demonstrated beneficial effects (Whurr et al., 1992; Robey, 1994; Holland et al., 1996; Bhogal et al., 2003; Meinzer et al., 2005), though in most cases, particularly when the lesion in the left hemisphere is large in size, patients do not fully recover (Lazar et al., 2010). Given the limited effectiveness of rehabilitative therapies in improving aphasia outcomes, other therapies, such as non-invasive brain stimulation by transcranial Direct Current Stimulation (tDCS) or repetitive Transcranial Magnetic Stimulation (rTMS), have been recently trialed in post-stroke language recovery. Findings, thus far, indicate that brain stimulation can enhance the effect of therapy in post-stroke aphasia (Monti et al., 2008; Baker et al., 2010; Fridriksson et al., 2011; Kang et al., 2011; Marangolo et al., 2011; You et al., 2011). However, these studies investigated the effect of non-invasive brain stimulation on aphasia outcomes by recruiting the left hemisphere. Given that this route of recovery is possible only when patients have a small left hemispheric lesion with at least some language areas remaining intact, finding a recovery route for patients with little intact left hemisphere language areas is an outstanding issue.

Vines et al. (2011) attempted to address this problem by suggesting a treatment option that enhances activity in the right hemisphere sensorimotor network for articulation. This treatment option is based on research that indicates the right hemisphere can (when engaged) assume language functions previously assigned to the left hemisphere (Bassow et al., 1989; Gainotti, 1993; Finger et al., 2003). In the Vines et al. (2011) study, six patients with moderate-to-severe non-fluent aphasia took part in two experimental phases that each involved three consecutive days of treatment. In the first phase of the study, patients were administered anodal-tDCS (a-tDCS) over the right Broca's homolog and Melodic Intonation Therapy (MIT). This form of tDCS has the potential to engage areas that are important for language recovery by delivering a weak electrical current to the cortex via a pair of electrodes to increase cortical excitability. MIT, on the other hand, engages language-capable areas in both hemispheres by training patients to intone (sing) phrases (Albert et al., 1973; Sparks and Holland, 1976; Zipse et al., 2012). In the second phase of the study, patients were administered sham-tDCS and MIT. Vines et al. (2011) found that a-tDCS applied to the right Broca's homolog in combination with MIT led to significantly greater improvements in fluency of speech compared to sham tDCS in combination with MIT. Vines et al. (2011) interpreted their findings to suggest that a-tDCS applied to Broca's homolog in the right inferior frontal gyrus can increase synaptic plasticity in brain areas already engaged by MIT and, therefore, augment the beneficial effects of the therapy.

The findings of Vines et al. (2011) are important because they imply a possible neurorehabilitive option for post-stroke aphasia patients who have little-to-no functional brain tissue remaining in the left inferior frontal gyrus and surrounding left hemisphere language areas. Specifically, the results of Vines et al. (2011) indicate that one of the only routes of recovery for these patients might involve the recruitment of right hemispheric language regions to compensate for damage in left hemispheric language regions. This proposal may at first seem to contradict the assumption that activity in the right hemisphere of post-stroke aphasia patients hinders language recovery (Rosen et al., 2000; for a review see Crosson, 2008). It is important to note, however, that this assumption does not imply that *any* right hemisphere activity following stroke is detrimental for language function. For example, Naeser et al. (2005) have shown that some right hemisphere activity may be beneficial for language recovery in persons with post-stroke aphasia (e.g., activity in right pars opercularis can aid in word-finding), whereas other right hemisphere activity may be detrimental for language recovery in persons with post-stroke aphasia (e.g., activity in right pars triangularis can hinder word-finding). Indeed, the existing literature indicates that activity in the right hemisphere plays a large role in the language recovery of patients with large left hemisphere lesions (Karbe et al., 1998; Cao et al., 1999; Heiss et al., 1999; Perani et al., 2003), such that if the right hemisphere is lesioned in patients with existing left hemisphere lesions then language function further deteriorates (Barlow, 1877; Gowers, 1887; Bassow et al., 1989; Crosson, 2007; Crosson et al., 2007). These results suggest that some right hemisphere mechanisms and structures are crucial for language recovery in people with post-stroke aphasia. Therefore, recruiting the right hemisphere through therapy and non-invasive brain stimulation may aid the recovery process.

Precisely which right hemisphere regions are important for aphasic compensation to occur is, however, difficult to ascertain from the results reported by Vines et al. (2011) because the stimulation technique they employed has limited spatial resolution (see Priori et al., 2009). For example, the active electrode was placed posterior to F8 of the International 10–20 System (assumed to correspond to Broca's homolog for each participant) and the other electrode was placed over the left supraorbital region. The placement of these electrodes on the scalp means that current flows throughout the brain between those two electrode sites and, consequently, nerve polarization may occur over that wide area. Furthermore, the tDCS electrodes used by Vines et al. (2011) had an area of 16.3 cm^2^, which, as noted by the researchers, may have extended stimulation from the assumed Broca's homolog area in the right inferior frontal gyrus into the anterior temporal cortex and ventral premotor cortex. Hence, due to poor focality of stimulation, it is unclear whether the benefit of a-tDCS and MIT on language function arises from stimulation of the right Broca's homolog (the area over which it was assumed that the active electrode was placed) or, more generally, the right inferior frontal gyrus and its surrounding right hemisphere areas (the area over which stimulation may have extended due to electrode size and placement).

The purpose of the present study was to investigate whether or not specifically stimulating the right Broca's homolog is sufficient to augment the positive effects of MIT on language function. To investigate this question we used high frequency rTMS as opposed to a-tDCS. The advantage of using rTMS as opposed to tDCS is that the focality of stimulation is more precise (stimulation can be limited to an area of about 25 mm^2^; Priori et al., 2009), hence Broca's homolog can be specifically targeted for stimulation. If the positive effect of a-tDCS and MIT on language function is indeed due to the stimulation of the right Broca's homolog then we should be able to replicate the findings of Vines et al. (2011) in the present study using rTMS applied to the right Broca's homolog. If, however, the positive effect of a-tDCS and MIT on language function is attributed to more generally stimulating the right inferior frontal gyrus and surrounding right hemisphere regions, then the findings of Vines et al. (2011) might fail to generalize to the present study. Although the present study is not the first to apply rTMS to the right Broca's homolog (e.g., Naeser et al., 2005, 2010, 2012; Garcia et al., 2013), it is the first to apply excitatory rTMS (as opposed to inhibitory or low frequency rTMS) to that region. Thus, the results of this investigation are important because they would indicate whether or not a combination of MIT and excitatory rTMS applied to the right Broca's homolog can improve post-stroke aphasia speech and language outcomes.

## Methods

### Participants

**Figure 1:**
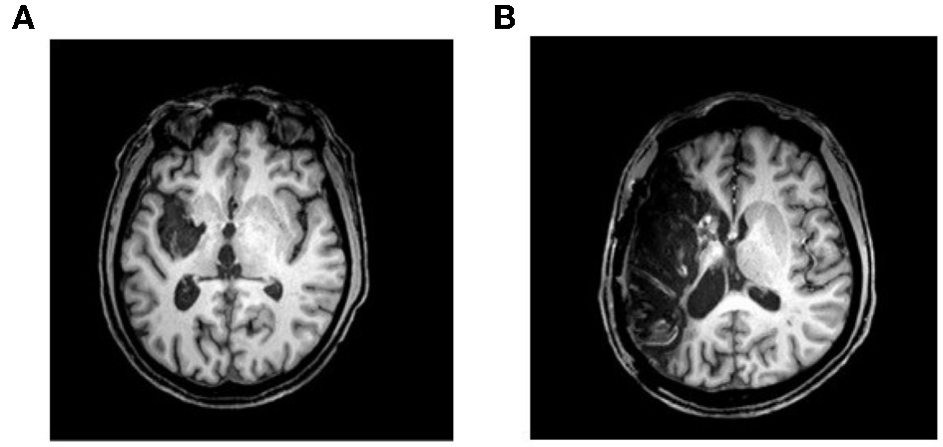
**Structural images of the left hemisphere lesions for participants (A) GOE and (B) AMC**. Participant AMC had a titanium mesh plate inserted into his left temporalis muscle following his stroke. The metal inserted into AMC's skull interfered with the MRI scan of that left region and, therefore, it is difficult to pinpoint the exact location of his lesion on the image.

Two males participants with non-fluent aphasia took part in this study. The protocol was approved by Macquarie University and the participants and their primary caregivers gave informed consent. The Western Aphasia Battery (WAB) was used to assess both participants' speech and language function. Participant GOE was 65 years old and 18 months post left fronto-temporal stroke (see Figure 1 for structural image). The WAB assessment classified GOE as having moderate non-fluent Broca's aphasia. GOE also presented with moderate right hemiparesis. Participant AMC was 49 years old and 20 months post left fronto-temporal stroke (see Figure 1). AMC had a titanium mesh plate inserted in his temporal region following the stroke. The WAB assessment classified AMC as having moderate-to-severe non-fluent Broca's aphasia with difficulties in auditory-verbal comprehension. AMC also presented with moderate right hemiparesis and moderate right visual neglect. GOE had knowledge of MIT through his group therapy sessions and AMC had been undergoing MIT for a year prior to participating in the study. The participants were both asked to halt all speech and language therapy for the duration of this study. GOE and AMC were right-handed prior to the stroke and both were native speakers of English.

### Language outcome measures

Language function was first assessed 1 week prior to commencing the study (baseline 1) and immediately before the first treatment session was conducted (baseline 2). After the study commenced, language testing was conducted after each treatment session and 1 week following the completion of the study. The language assessment tasks included the automatic production of verbal sequences (i.e., counting from 1 to 21, reciting the days of the week, reciting the months of the year, reciting the alphabet, and listing as many animals as possible within 1 min) and a phrase repetition task. There were 51 phrases in the phrase repetition task (see Table A1 for phrases). These phrases had no more than 4 syllables (e.g., “I love you,” “Goodbye,” “Pass the salt,” “Hi”). The verbal output for both the phrase repetition and verbal fluency tasks was recorded and later transcribed.

The phrases used during the repetition task were divided into 3 lists of 17 phrases that were matched for utterance length, syntactic structure, and frequency/imageability. Each of these lists was randomly assigned to one of 3 conditions for each participant: rTMS-treated, sham-rTMS treated, and untreated. The rTMS-treated list was used by the clinician to provide MIT following rTMS, whilst the sham-rTMS treated list was used by the clinician to provide MIT following sham-rTMS. The untreated list was not practiced during MIT. The phrases practiced during MIT were, therefore, a subset of the phrases presented in the repetition task for language assessment.

### fMRI data acquisition and preprocessing

Anatomical and functional images were collected at Macquarie University Hospital, Sydney, using a 3 Tesla Siemens MagnetonVerio scanner with a 32-channel head coil. First, an alignment scan was performed for head position adjustments so that the AC-PC reference line was as close as possible to the vertical axis of the scanner. Second, a T1-weighted anatomical image was obtained using the following parameters: *TR/TE* = 2000 ms/900 ms, FOV = 250 mm, flip angle = 9 degrees and voxel size = 1 mm^3^. Third, T2-weighted functional images were acquired using the following parameters: *TR/TE* = 3000 ms/32 ms, FOV = 240 mm, flip angle = 80 degrees, gap = 0.5, number of slices = 78 and voxel size = 2.5 mm^2^. The order of acquisition was ascending, interleaved. All sessions were started with 2 dummy scans. Brain activation was measured by adopting the blood oxygenation level-dependent (BOLD) effect with optimal contrast (Ogawa et al., 1990). The collected images were preprocessed using the Statistical Parametric Mapping software (SPM8; http://www.fil.ion.ucl.ac.uk/spm). Corrections were made for the time delay in acquisition of the different slices, and the images were realigned onto the mean image for head-motion correction and spatially normalized into a standard stereotaxic space with voxel size of 3 mm^3^ using the Montreal Neurological Institute (MNI) template. A spatial smoothing filter was employed for each volume by using an isotropic Gaussian kernel (FWHM = 6 mm).

### fMRI overt language tasks

Participants underwent an fMRI session one week prior to the start of the study and 1 week following the end of the study. The scans were used to localize Broca's homolog for targeted stimulation in the rTMS portion of the treatment and to track any functional cortical reorganization occurring during the study. Participants were asked to complete two tasks in the scanner: an automatic speech task and a naming/reading task. All stimuli were projected onto a screen and viewed through a mirror mounted on the head coil. Verbal responses were recorded using a FOMR-III MRI compatible microphone (Optoacoustics Ltd) attached to the head coil in the scanner. The overt responses were also transcribed at the time of scanning. Stimulus presentation and recording of the verbal responses was controlled by Presentation (Neurobehavioral Systems Inc). The participants were trained on each task prior to the fMRI sessions. A block-design was used to present the tasks in the scanner because it permits an examination of overt speech in participants with aphasia despite the false-starts and hesitations found in such participants (Martin et al., 2009). Such designs also have excellent statistical power (Aguirre et al., 1997).

### Automatic speech task

**Figure 2:**
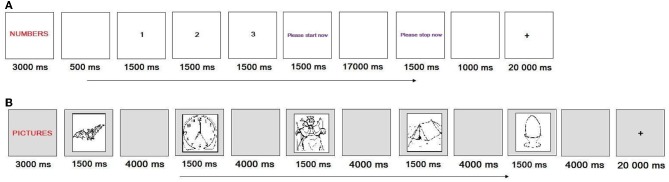
**The sequence of displays for (A) the automatic speech task, and (B) naming/reading task that GOE and AMC were asked to complete in the scanner**. **(A)** represents a trial in which the participant had to count from 1 to 21 in the right order, whereas **(B)** represents a block of trials in which the participant had to name pictures in a series. +, Fixation.

In the automatic speech task, participants were required to count from 1 to 21 and recite the days of the week, months of the year and the alphabet. When completing this task (see Figure 2A), participants saw a category label appear on the screen for 3 s (e.g., NUMBERS) followed by three examples of the category sequence (e.g., 1, 2, 3) that appeared for 1.5 s each. Participants were given 20 s to recite (in correct order) each item in the category. The order of the categories was randomized. There were two runs of this task. Each run consisted of 4 trials that were separated by a 20 s fixation.

### Naming/reading task

In the naming/reading task, participants were asked to name a picture or read a word. There were 10 items that appeared as both pictures and words. The items were selected from The International Picture Naming Project database (http://crl.ucsd.edu/experiments/ipnp/; Szekely et al., 2004) based on length (one syllable), frequency (high) and percent of dominant response (100% of participants produced the dominant name). When completing this task (see Figure 2B), participants saw an instruction label appear on the screen for 3 s (e.g., PICTURE or WORD) followed by 5 items (e.g., king, bat, leaf, map, egg) that each appeared for 1.5 s. Participants were given 4 s to name each picture or read each word. The pictures appeared in a separate block to the words. Block order was counterbalanced. Each block was separated by a 20 s fixation. There were four blocks in each run. Participants completed two runs.

### rTMS treatment protocol

Both GOE and AMC participated in two phases of the TMS protocol: rTMS and sham-TMS. The order of the two phases was counterbalanced across participants and was separated by a 1-week break. GOE first went through the rTMS phase, whereas AMC first went through the sham-TMS phase. Each phase consisted of three treatment sessions that were separated by 3 days (see Table 1 for treatment schedule).

**Table 1:** **Schedule of treatment for: (A) participant GOE and (B) participant AMC**.

**(A) Treatment schedule for GOE**					
**Day 1**	**Day 2**	**Day 3**	**Day 4**	**Day 5**	**Day 6**
fMRI 1 and Baseline 1					
**Day 7**	**Day 8**	**Day 9**	**Day 10**	**Day 11**	**Day 12**
Baseline 2 and rTMS + MIT post-test				rTMS + MIT post-test	
**Day 13**	**Day 14**	**Day 15**	**Day 16**	**Day 17**	**Day 18**
	rTMS + MIT post-test				
**Day 19**	**Day 20**	**Day 21**	**Day 22**	**Day 23**	**Day 24**
		sham + MIT post-test			
**Day 25**	**Day 26**	**Day 27**	**Day 28**	**Day 29**	**Day 30**
sham + MIT post-test			sham + MIT post-test		
**Day 31**	**Day 32**	**Day 33**	**Day 34**	**Day 35**	
				fMRI 2 follow up	
**(B) Treatment schedule for AMC**					
**Day 1**	**Day 2**	**Day 3**	**Day 4**	**Day 5**	**Day 6**
fMRI 1 and Baseline 1					
**Day 7**	**Day 8**	**Day 9**	**Day 10**	**Day 11**	**Day 12**
Baseline 2 and sham + MIT post-test				sham + MIT post-test	
**Day 13**	**Day 14**	**Day 15**	**Day 16**	**Day 17**	**Day 18**
	sham + MIT post-test				
**Day 19**	**Day 20**	**Day 21**	**Day 22**	**Day 23**	**Day 24**
		rTMS + MIT post-test			
**Day 25**	**Day 26**	**Day 27**	**Day 28**	**Day 29**	**Day 30**
rTMS + MIT post-test			rTMS + MIT post-test		
**Day 31**	**Day 32**	**Day 33**	**Day 34**	**Day 35**	
				fMRI 2 follow up	

GOE began with rTMS treatment whereas AMC began with sham-rTMS treatment.

Surface electromyography (EMG) electrodes were placed over the first dorsal interosseous (FDI) of the left hand. Single-pulse TMS was performed to establish active motor threshold (AMT) using a Magstim Rapid stimulator. Stimulation was conducted using a 70 mm figure-8 coil. The coil was placed with the handle pointing occipitally at an angle of approximately 45° to the mid-sagittal line over the primary motor cortex in the right hemisphere at the optimal site for obtaining an MEP in the FDI. After AMT was established, intermittent theta-burst stimulation (iTBS), a form of high frequency rTMS, was performed using Magstim Rapid2 with intensity set at 80% of AMT. The iTBS consisted of bursts of 3 pulses at 50 Hz given every 200 ms in 2 s trains, repeated every 10 s over 200 s for a total of 600 pulses. Coil position for iTBS was determined by examining the T2-weighted MRI scans of each participant. The target site for stimulation was selected by identifying a peak of frontal activity (separately for each participant) within the anatomical territory of pars triangularis (GOE) or pars opercularis (AMC) in the right inferior frontal gyrus. This peak voxel activity was elicited by the automatic speech task. The surface distance measurements method was used to identify the location of the selected region on the head of each participant (Weiduschat et al., 2009). The same iTBS procedure was used in the sham-rTMS phase, but a sham TMS coil was substituted for the real TMS coil. The sham coil mimics a real TMS coil by producing the same clicking sound, but, unlike a real TMS coil, the sham coil does not cause any change in neural activity. Each rTMS session lasted 5-min and was followed by a 5-min break. Participants completed a 40-min MIT session after the break, which was administered by a clinician. Participants and the clinician were blind to stimulation condition. The MIT session followed the format of a typical MIT protocol with phrases trained using a hierarchical series of steps (Norton et al., 2009). Each phrase was intoned on the minor third interval with regular syllable durations of 1 s.

## Results

### Language outcome data

#### Phrase repetition task

**Figure 3:**
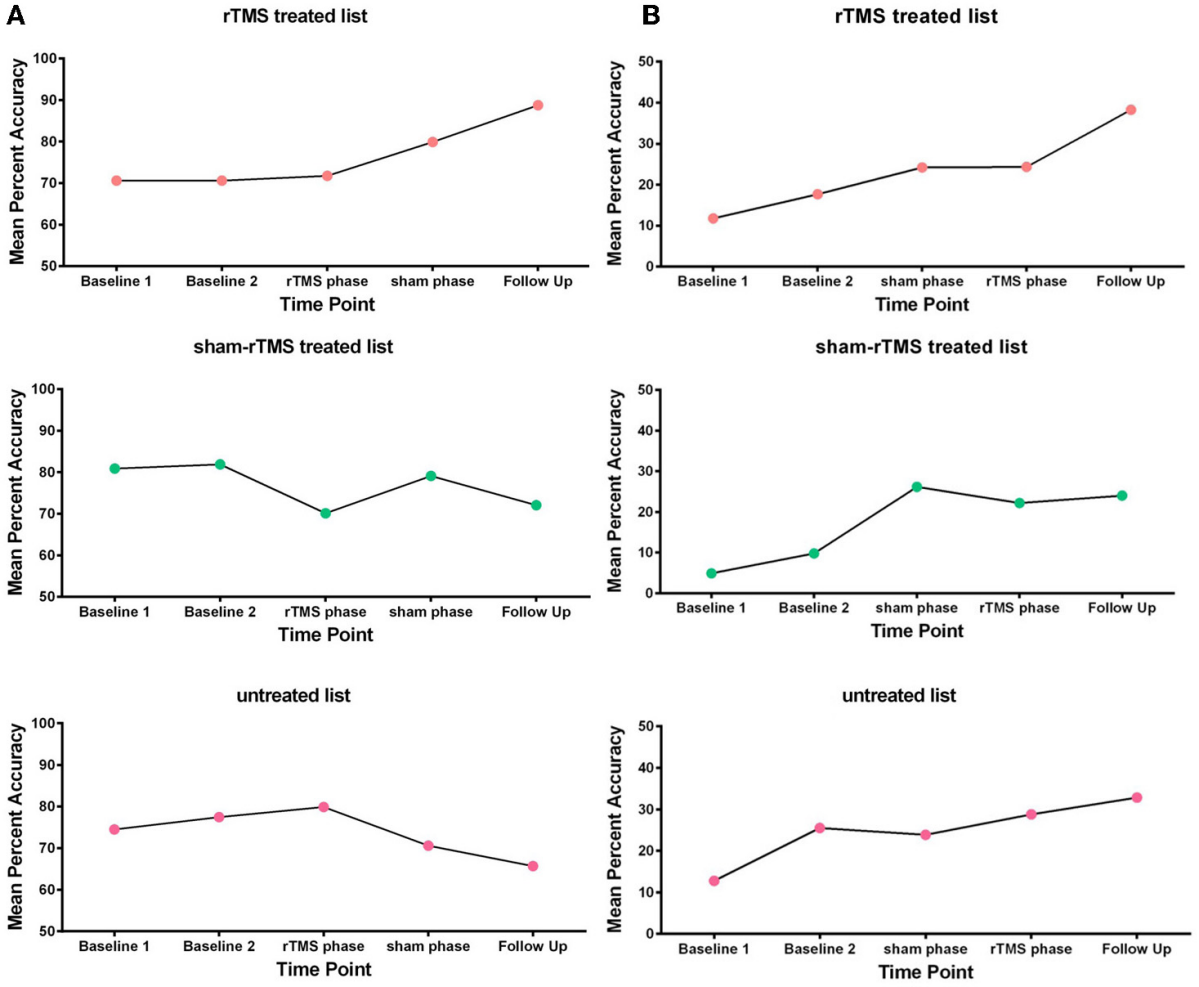
**Mean percent accuracy of phrase repetition at baseline, during the treatment phases, and at the follow up session for (A) participant GOE and (B) participant AMC**.

Performance on the phrase repetition task was measured by the number of words correctly repeated in each phrase. First, those scores were used to calculate mean percent accuracy of phrase repetition at baseline and throughout the treatment phases (rTMS phase, sham-rTMS phase and follow-up). Second, for each of the three phrase lists, an examination of whether or not there was significantly greater improvement during the treatment phase (rTMS phase, sham-rTMS phase, follow-up) compared to the pre-treatment baseline (baseline 1, baseline 2) was conducted using a one-sample *t*-test on the sum (across the five testing points) of the weighted scores for each phrase list. The null hypothesis was that there would be no change across the five testing points. For GOE (see Figure 3A), there was significantly greater improvement in the treatment phase for the rTMS treated list, *t*_(16)_ = 2.08, *p* = 0.02, but no significant improvement for either the sham-rTMS treated list, *t*_(16)_ = 1.15, *p* = 0.50, or the untreated list, *t*_(16)_ = 1.40, *p* = 0.91. However, as indicated in Figure 3A, GOE's improvement on the rTMS treated list appears to be delayed, such that the treatment gains only became evident at the later testing points. AMC, in contrast, showed a clear practice effect across the pre-treatment baselines, but there was no evidence of greater improvement during the treatment phase as compared to baseline (rTMS treated list, *t*_(16)_ = 0.18, *p* = 0.43; sham treated list, *t*_(16)_ = 0.75, *p* = 0.77; untreated list, *t*_(16)_ = 1.09, *p* = 0.85; see Figure 3B).

#### Verbal fluency task

**Figure 4:**
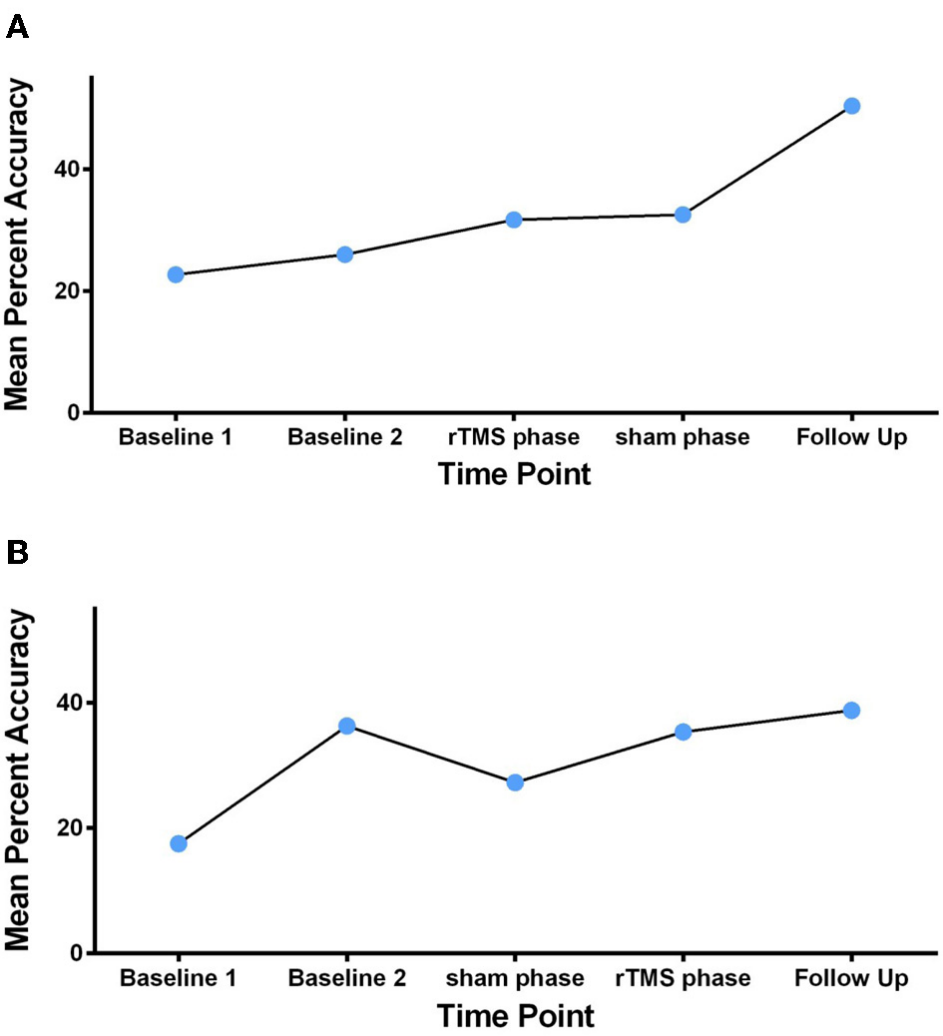
**Mean percent accuracy of verbal fluency at baseline, during the treatment phases, and at the follow up session for (A) participant GOE and (B) participant AMC**.

Performance on the verbal fluency task was measured by the percentage of items produced in the correct order for each category. Those scores were used to calculate mean percent accuracy in the verbal fluency task at baseline, throughout the sham-rTMS and rTMS phases, and at the 1-week follow-up session. The results were analyzed using the same statistical method as the phrase repetition task, although it should be noted that power is low with only 5 fluency measures. Nevertheless, GOE's fluency showed a trend to significantly more improvement during the treatment phase than the baseline phase, *t*_(4)_ = 1.59, *p* = 0.09 (see Figure 4A). AMC did not show any evidence of significantly greater improvement during the treatment phase, *t*_(4)_ = 0.84, *p* = 0.23 (see Figure 4B).

### fMRI overt language data

#### Automatic speech task

The automatic speech task was scored using the same procedure as the verbal fluency task. The results revealed that GOE's mean percent accuracy in the automatic speech task improved from 14% in the pre-treatment session to 18% in the post-treatment session (see Table 2). Similarly, AMC's mean percent accuracy in the automatic speech task improved from 3% in the pre-treatment session to 11% in the post-treatment session.

**Table 2:** **Number of items correctly produced by (A) GOE and (B) AMC during the automatic speech task in both the pre and post treatment fMRI sessions**.

	**Pre-treatment**	**Post-treatment**
**(A) GOE**		
	**RUN 1**	**RUN 1**
Day	0	4
Month	0	0
Number	0	0
Letter	6	6
	**RUN 2**	**RUN 2**
Day	2	0
Month	0	7
Number	10	0
Letter	3	1
**(B) AMC**		
	**RUN 1**	**RUN 1**
Day	0	0
Month	0	0
Number	3	12
Letter	1	4
	**RUN 2**	**RUN 2**
Day	0	0
Month	0	0
Number	1	4
Letter	0	0

#### Naming/reading task

Performance on the naming/reading task was measured by the percent of items correctly read or named. The results indicated that GOE's percent accuracy in the naming/reading task reduced from 80% in the pre-treatment session to 70% in the post-treatment session. Furthermore, AMC's percent accuracy in the naming/reading task also reduced from 7.5% in the pre-treatment session to 0% in the post-treatment session.

### fMRI functional data

Region-of-interest (ROI) analyses were conducted to compare pre- and post–treatment fMRI sessions for each participant^1^. Specifically, this analysis was used to examine the effect of treatment on activation in the left Broca's area and right Broca's homolog. This method of analyzing fMRI activation is recommended when making statistical comparisons from one session to another in an individual participant as opposed to a group of participants because it avoids threshold effects (e.g., Peck et al., 2004; Martin et al., 2009). The ROI analyses were performed with the Marsbar toolbox in SPM8 (Brett et al., 2002; http://marsbar.sourceforce.net) and Broca's area in the inferior frontal gyrus was defined by masks BA44 and BA45 taken from Anatomy Toolbox (Eickhoff et al., 2005; www.fz-juelich.de/ime/spm_anatomy_toolbox). These masks correspond to the pars opercularis and pars triangularis, respectively. For each of those ROIs, individual measures of mean effect size for the active (speaking) condition relative to the fixation condition were obtained for each participant. Statistical analysis of mean activation was then conducted separately for each ROI by entering the SPM contrast images into a paired *t*-test to determine differences in activation before and after treatment. Each contributing contrast was set at *p* < 0.05, uncorrected given that our data analysis was hypothesis-driven (Neumann et al., 2005).

#### Automatic speech task

**Figure 5:**
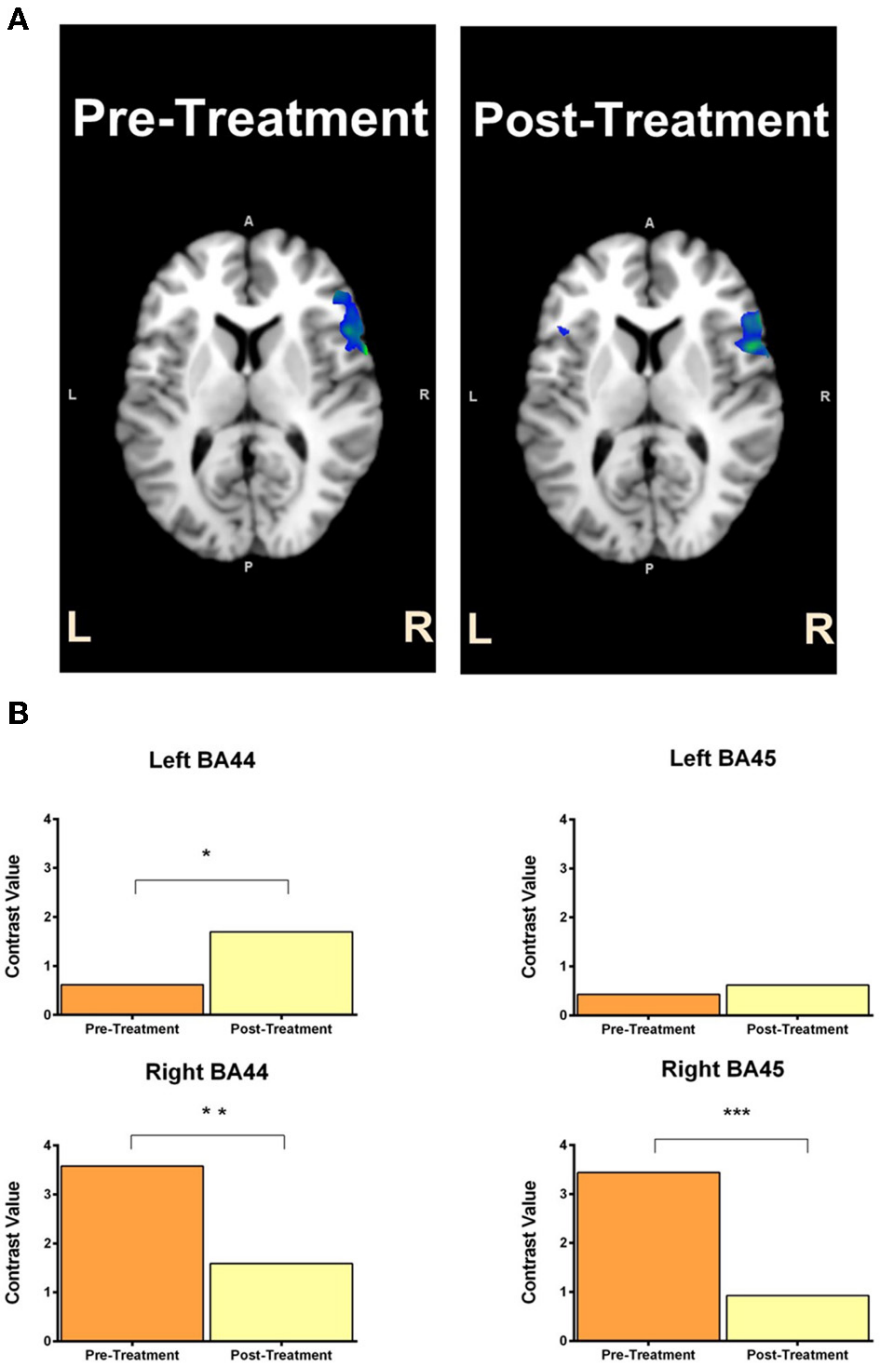
**GOE's statistical activation within the ROI (left Broca's area and right Broca's homolog) is shown in panel (A) for the contrast of speaking vs. fixation in the automatic speech task**. The reconstructed ROIs for pre-treatment and post-treatment are projected on the axial anatomic background (MNI) reference brain. Panel **(B)** reflects contrast values related to ROI activity in the pre-treatment and post-treatment scans for GOE (with 95% CI) in the automatic speech task. Contrast value refers to the value of ß in the analyzed contrasts of pre-treatment >post-treatment (top) and post-treatment > pre-treatment (bottom). Asterisk ^*^indicates *p* < 0.05 uncorrected, ^**^reflects *p* < 0.01 uncorrected and ^***^denotes *p* < 0.001 uncorrected.

**Figure 6:**
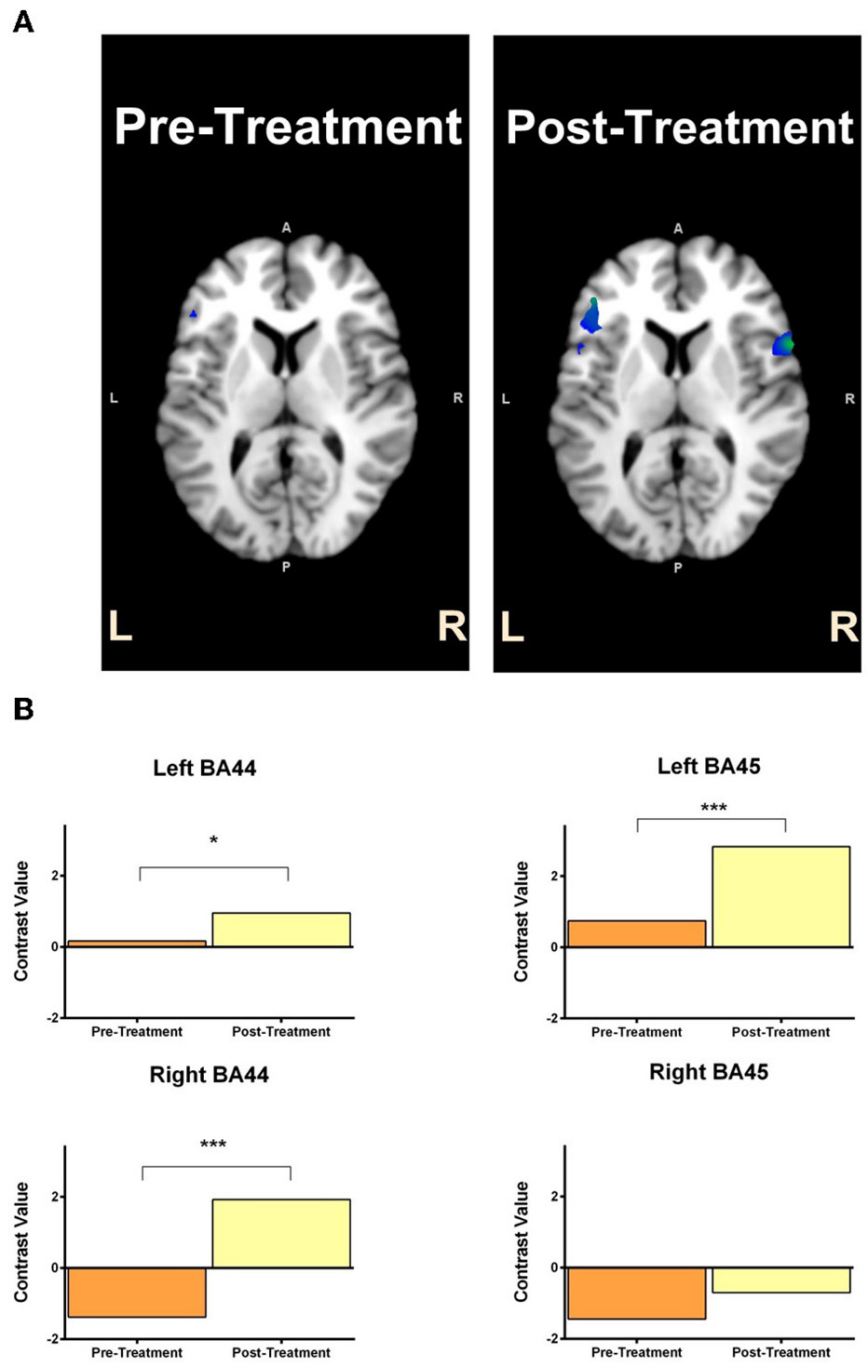
**AMC's statistical activation within the ROI (left Broca's area and right Broca's homolog) is shown in panel (A) for the contrast of speaking vs. fixation in the automatic speech task**. The reconstructed ROIs for pre-treatment and post-treatment are projected on the axial anatomic background (MNI) reference brain. Panel **(B)** reflects contrast value scores related to ROI activity in the pre-treatment and post-treatment scans for AMC (with 95% CI) in the automatic speech task. Contrast value refers to the value of ß in the analyzed contrasts of pre-treatment >post-treatment (top) and post-treatment > pre-treatment (bottom). Asterisk ^*^indicates *p* < 0.05 uncorrected, ^**^reflects *p* < 0.01 uncorrected and ^***^denotes *p* < 0.001 uncorrected.

For GOE, the ROI analyses revealed significant activity increase from pre to post treatment in the left BA44, *t* = 1.79, *p* < 0.05, during the automatic speech task (Figure 5). This pre to post treatment ROI difference was also related to significant activity decrease in the right BA44, *t* = 2.92, *p* < 0.01, and right BA45, *t* = 3.36, *p* < 0.001, during the automatic speech task (Figure 5). For AMC, the ROI analyses revealed a significant increase in activity from pre to post treatment in the left BA44, *t* = 1.77, *p* < 0.05, left BA45, *t* = 3.51, *p* < 0.001, and right BA44, *t* = 4.92, *p* < 0.001, during the automatic speech task (Figure 6).

#### Naming/reading task

The comparison between the pre and post treatment sessions for both GOE and AMC revealed no differences in brain activity.

## General discussion

The aim of the present study was to investigate whether or not stimulating right Broca's homolog using high frequency rTMS, which limits stimulation to a small area, can enhance the benefits of MIT on post-stroke recovery. Indeed, Vines et al. (2011) recently observed that a combination of MIT and a-tDCS applied to the right inferior frontal gyrus can produce greater benefits in post-stroke aphasia compared to MIT alone. However, it was unclear which right hemisphere areas were responsible for the beneficial effects of a-tDCS and MIT on language function. Vines et al. (2011) could not resolve this issue because the method of non-invasive brain stimulation that they used (tDCS) has poor spatial resolution. On the one hand, it is possible that the benefit of a-tDCS and MIT on language function resulted from stimulating, in particular, the right Broca's homolog. On the other hand, it is possible that the benefit of a-tDCS and MIT on language function resulted from stimulating, more generally, the right inferior frontal gyrus and surrounding right hemisphere areas. We find several interesting patterns of results in the present preliminary study. These results indicate the importance of further investigating the combined effect of MIT and high frequency rTMS applied to the right Broca's homolog on speech and language function.

Behaviorally, the effects of treatment were modest, which is not surprising given that we had only three MIT sessions in each TMS phase. AMC showed no statistically reliable evidence of benefits from the treatment, over and above practice effects, in both the phrase repetition and verbal fluency tasks. GOE, in contrast, showed evidence of significant benefits from treatment in the phrase repetition task. Specifically, and more importantly, GOE's benefit from treatment was restricted to those phrases that received MIT in combination with TMS. This result suggests that TMS applied to Broca's homolog in the right hemisphere may enhance the effect of MIT for at least some individuals with aphasia. It is important to note, however, that GOE improved on the rTMS treated list during the later treatment sessions, which is a pattern consistent with the suggestion that the therapeutic effect of rTMS may develop some time after the stimulation period (Hoogendam et al., 2010; Badawy et al., 2012; for a review see Mally, 2012). It is unlikely that GOE's improvement on the rTMS treated list is due to a practice effect as there was no evidence of greater improvement (over and above changes in the baseline phase) for the sham and untreated lists. In the verbal fluency task, moreover, GOE showed improvement, albeit modest, over the course of the treatment. This pattern of results is consistent with Vines et al. (2011) in showing that a combination of non-invasive brain stimulation (here rTMS instead of tDCS) and MIT can, at least for some participants, also improve verbal fluency. Hence, although we caution that the results from our case studies should not be over interpreted, our findings do indicate that MIT and high frequency rTMS applied to the right Broca's homolog can have positive effects on post-stroke aphasia outcomes for some people with non-fluent aphasia.

In addition to the behavioral outcomes, we also observed that the pattern of cortical activity during speaking modestly changed over the course of treatment for both participants. GOE showed an increase in left Broca's activation (BA44) from the pre-treatment to post-treatment sessions. This increase in left hemisphere activation was also accompanied by a decrease in right Broca's homolog activation (BA44 and 45). These results indicate that stimulating the right Broca's homolog did not induce long-lasting neural changes in the expected direction; that is, greater right Broca's homolog activity. Rather, this shift in activation from the right hemisphere to the left hemisphere is consistent with studies that show decreased right hemisphere activation alongside increased left hemisphere activation following MIT (Laine et al., 1994; Breier et al., 2010). AMC, in contrast, showed increased activation in the left Broca's area (BA44) and right Broca's homolog (BA44 and 45) from the pre-treatment to post-treatment sessions. This pattern of results is interesting as it suggests that treatment increased the extent of activation in both AMC's left and right hemisphere language areas. AMC's neurophysiological results are at odds with our expectation that stimulating the right Broca's homolog may increase neural activity in (only) that region. This result is only partially consistent with the pattern of activation shown by Breier et al. (2010), which suggested increased left hemisphere activation following MIT treatment, and the opposing pattern of activation found by Schlaug et al. (2010), which suggested increased right hemisphere activation following MIT treatment. AMC's pattern of activation is, however, consistent with results of a study that showed increased bilateral activation following anomia treatment (Rochon et al., 2010). It is important to mention that we observed these neural changes for both participants only in the automatic speech task, but not the naming/reading task. It is possible that as MIT training is highly repetitive and becomes automatized, neural changes are restricted to mechanisms involved in automatic speech rather than (the more conscious) propositional speech, which is required for the naming/reading task. The imaging results of GOE and AMC nevertheless indicate that our treatment may have induced neural activity changes in the left Broca's area and right Broca's homolog.

There are several points to consider before applying the findings of the present study to a more general population of non-fluent aphasia patients. First, we examined the effect of rTMS in combination with MIT on only two participants. Our participants varied in age (16 years difference), aphasia severity and experience with MIT, all of which may affect their potential for speech recovery and account for any differences in findings between them. Second, in contrast to Vines et al. (2011), our treatment sessions were not conducted consecutively, but rather were separated by 3 days. This treatment schedule may have, therefore, resulted in less cumulative build up of rTMS-induced plasticity and, in turn, reduced the potential for non-invasive brain stimulation to augment the benefits of MIT on language function. Future studies should compare the effect of consecutive vs. dispersed treatment sessions on language function, and, moreover, increase the number of treatments in each series. Third, the neurophysiological changes that we observed may be due to the effect of MIT on the bilateral neural network for language, the change in performance level from pre-treatment to post-treatment due to practice, or both. As a result, it would be beneficial for future studies to utilize an event-related design as opposed to a blocked-design to examine patterns of activation for correct vs. incorrect responses. Alternatively, measuring activation across two occasions prior to treatment would help ensure that any changes in neural activity are specifically attributed to the effect of treatment as opposed to variability in performance or practice effects. We also recommend including a no-treatment control group that is tested over the same interval to examine whether or not the behavioral and imaging data change over time. This comparison between the no-treatment group and the treatment group would ensure that any neurophysiological changes observed are not due to normal variation in activation patterns. Fourth, the difference in treatment outcome between GOE and AMC could be due to the difference in which region was stimulated (right pars triangularis vs. right pars opercularis, respectively). It would, therefore, be beneficial for future studies to examine the relationship between each stimulation area in the right Broca's homolog and language function. This examination could also compare the effect of excitatory rTMS to inhibitory rTMS applied to the right Broca's homolog on language function. Such a comparison would ascertain the correlation between performances on specific language tasks and right Broca's homolog activity.

## Conclusion

Previous studies have suggested that non-fluent aphasia patients with large left hemisphere lesions may recover speech ability when there is interhemispheric transfer of language function. The purpose of this preliminary study was to examine whether or not interhemispheric transfer could be facilitated by engaging the contralesional right hemisphere using high frequency rTMS applied to the right Broca's homolog and MIT. We found that one of our participants, GOE, made modest improvements in verbal fluency and the repetition of phrases treated with MIT in combination with rTMS applied to the right Broca's homolog. The second participant, AMC, did not show any improvement as a result of treatment. Given the pattern of results, it is likely that the gains made by GOE in the phrase repetition and verbal fluency tasks were a result of rTMS augmenting the benefits of MIT. The fMRI data, moreover, indicate that both participants showed neural activity changes in the right and left hemisphere language networks following treatment, which, at least in part, was linked to better performance on automatic speech tasks. These neural changes are most likely due to the effect of therapy. Our two case studies provide preliminary information toward understanding the effect of excitatory rTMS applied to the right Broca's homolog and MIT on the language function of post-stroke aphasics. This treatment protocol has potential for participants with large left hemisphere lesions and should be further tested in studies assessing the short and long term efficacy of non-invasive brain stimulation and speech therapy on post-stroke aphasia speech and language recovery.

### Conflict of interest statement

The authors declare that the research was conducted in the absence of any commercial or financial relationships that could be construed as a potential conflict of interest.

## Footnotes

^1^We also conducted a whole-brain analysis of the functional data (see Tables S1–S4). Voxel thresholds for all contrasts were set to p <0.05 family-wise error corrected (FWE) with a cluster threshold of k > 10. The analysis revealed increased activation in the left hemisphere language areas, such as the middle temporal gyrus, inferior temporal gyrus, fusiform gyrus, middle frontal gyrus, superior frontal gyrus and precuneus following treatment. The right prefrontal cortex and right cingulate gyrus also increased in activity following treatment.

## Supplementary material

Table S1 **Summary of GOE's fMRI activation patterns for the main contrasts: pre-treatment > post-treatment and post-treatment > pre-treatment in the automatic speech task**.

Table S2 **Summary of AMC's fMRI activation patterns for the main contrasts: pre-treatment > post-treatment and post-treatment > pre-treatment in the automatic speech task**.

Table S3 **Summary of GOE's fMRI activation patterns for the main contrasts: (A) pre-treatment > post-treatment and post-treatment > pre-treatment in the pictures block and (B) pre-treatment > post-treatment and post-treatment > pre-treatment in the words block**.

Table S4 **Summary of AMC's fMRI activation patterns for the main contrasts: (A) pre-treatment > post-treatment and post-treatment > pre-treatment in the pictures block and (B) pre-treatment > post-treatment and post-treatment > pre-treatment in the words block**.

## Appendix

**Table A1:** **Phrases used during the phrase repetition task**.

**Set A**	**Set B**	**Set C**
We love you	You know him	I like her
I am fine	She is well	We are good
We want fruit	I need help	He needs time
Goodbye	Good day	Goodnight
She wants a pen	He needs a drink	I carry a bag
I am warm	You are hot	She is cold
Packet of chips	Carton of juice	Bottle of milk
Pass the salt	Bring the bread	Use the knife
Bye	Hi	Ta
Buy sugar	Dust pepper	Pour coffee
I'll come	He's back	We'll see
Read a book	Write a note	Send a card
Let's go	Let's leave	Let's stay
Go to him	Come to them	Talk to me
You are burnt	I am bored	He is tired
Yes, please	No, thanks	Too bad
Dinner time	Supper time	Breakfast time

Two sets from this list were also used for MIT training during the treatment period. GOE was trained on sets A and B during the rTMS phase and the sham-rTMS phase, respectively. AMC was trained on sets B and C during the rTMS phase and the sham-rTMS phase, respectively.
