# Supplementary material for: Augmenting melodic intonation therapy with non-invasive brain stimulation to treat impaired left-hemisphere function: two case studies
Source: Front Psychol. 2014 Feb 4;5:37. doi: 10.3389/fpsyg.2014.00037 (PMC3912988; doi:10.3389/fpsyg.2014.00037)
Supplement: Table S1 — Summary of GOE's fMRI activation patterns for the main contrasts: pre-treatment > post-treatment and post-treatment > pre-treatment in the automatic speech task. [file DataSheet1.PDF]

Supplementary Tables
Augmenting Melodic Intonation Therapy with non-invasive brain stimulation to treat impaired left-hemisphere function: two case studies

Shahd Al-Janabi[1,2], Lyndsey Nickels[1,2,3], Paul F Sowman[1,2] , Hana Burianová[1,4], Dawn L Merrett[5]and William F Thompson[1, 6]

[1]ARC Centre of Excellence in Cognition and its Disorders, Macquarie University, Sydney, Australia

[2] Department of Cognitive Science, Macquarie University, Sydney, Australia

[3]NHMRC Centre of Clinical Research Excellence in Aphasia Rehabilitation

[4]Centre for Advanced Imaging, The University of Queensland, Queensland, Australia

[5]Melbourne School of Psychological Sciences, The University of Melbourne, Melbourne, Australia

[6]Department of Psychology, Macquarie University, Sydney, Australia

Shahd Al-Janabi
Department of Cognitive Science
Macquarie University
Sydney, NSW
Australia 2109
Email: shahd.al-janabi@mq.edu.au
Fax: (612) 9850 6059

Supplementary Table 1

Automatic Speech Task

Region	Hemi	Pre-treatment > Post-treatment					
		BA	*k*	*x*	*y*	*z*	*T*
Lingual Gyrus	L	18	13	-16	-80	-4	5.7
Middle frontal gyrus	R	9	120	36	28	28	6.1
				34	36	32	5.9
Inferior frontal gyrus	R	47	22	24	26	-12	6.2
Claustrum	L		18	-24	22	-8	5.5
Inferior parietal lobule	R	40	14	66	-28	48	5.7

Region	Hemi	Post-treatment > Pre-treatment					
		BA	*k*	*x*	*y*	*z*	*T*
Thalmus	R		55	12	-30	4	6.6
Parahippocampus	R	30	55	12	-38	8	5.6
Middle frontal gyrus	L	10	10	-32	38	4	5.3
Cingulate gyrus	R	23	33	8	-44	26	6.7

L = left, R = right, Hemi = hemisphere; BA = Brodmann Area; *k* = cluster extent; *x/y/z* = MNI coordinates and *T*- values refer to maximally activated voxel for each significant cluster, voxel threshold $p < 0.05$ FWE-corrected, $k > 10$, anatomic structures have been determined using NITRC Mango (Kochunov et al., 2002)

Supplementary Table 2

Automatic Speech Task

Region	Hemi	Pre-treatment > Post-treatment					
		BA	*k*	*x*	*y*	*z*	*T*
Parahippocampus	R	35	109	34	0	-26	7.5
Fusiform gyrus	R	20	109	44	-6	-28	6.2
Middle temporal gyrus	L	21	45	-66	-30	-14	6.5
			45	-64	-18	-16	5.8
Anterior-superior Insula	R	13	13	-30	18	-16	5.7
Anterior cingulate	R	32	68	16	50	-14	6.5
Middle frontal gyrus	R	11	68	24	44	-14	5.6
Inferior frontal gyrus	R	47	52	38	20	-26	6.5
			52	30	14	-24	5.1
			10	40	30	-20	5.9

Supplementary Table 2

Region	Hemi	Post-treatment > Pre-treatment					
		BA	*k*	*x*	*y*	*z*	*T*
Cuneus	L	19	24	-24	-86	36	6
Lingual gyrus	R	18	38	6	-76	4	5.6
Culmen	L		20528	-26	-30	-22	11.9
Parahippocampus	L	28		-20	-22	-20	11.9
		30	38	-20	-46	2	6.3
	R	28	1169	18	-40	0	10.1
Thalmus	R		159	24	-18	4	8.2
	L			12	-22	2	6.9
	L		15	-22	-22	10	6
Superior temporal gyrus	L	38	289	-38	4	-24	7.8
Middle temporal gyrus	L	21		-46	-8	-24	7.4
				-54	-6	-22	7.2
		39	209	-26	-52	24	6
Cingulate gyrus	L	31		-18	-52	28	7.1
	R	23	16	8	-10	34	6
Precentral frontal gyrus	R	4	20528	58	-4	40	14.3
			18	38	-12	46	6.7
	L		1934	-26	-16	68	8.9
			16	-52	-8	44	6.8
Paracentral frontal gyrus	R	4	1169	16	-32	60	9.7
Superior frontal gyrus	L	8	48	-16	40	44	6.8
Middle frontal gyrus	L	8	46	-24	14	38	6.7
		9	10	-32	24	22	6.1
Precuneus	R	7	1169	16	-42	54	9.2
	L		1934	-6	-46	68	9.2
		39	209	-32	-62	40	6.4
Postcentral parietal gyrus	L	3	1934	-22	-30	68	9.6
		43	18	-64	-14	18	5.8
Inferior parietal lobule	L	40	42	-62	-32	24	7.3
				-64	-24	26	7.2
			30	-44	-50	48	5.5
Supramarginal gyrus	L	40	11	-50	-44	38	6.1
Superior parietal gyrus	L	7	30	-38	-54	54	5.8

L = left, R = right, Hemi = hemisphere; BA = Brodmann Area; *k* = cluster extent; *x/y/z* = MNI coordinates and *T* - values refer to maximally activated voxel for each significant cluster, voxel threshold $p < 0.05$ FWE-corrected, $k > 10$, anatomic structures have been determined using NITRC Mango (Kochunov et al., 2002)

Supplementary Table 3

a)

Naming/Reading Task (pictures block)

**Region**	**Hemi**	**Pre-treatment > Post-treatment**					
		**BA**	***k***	***x***	***y***	***z***	***T***
No significant cluster							

**Region**	**Hemi**	**Post-treatment > Pre-treatment**					
		**BA**	***k***	***x***	***y***	***z***	***T***
Caudate nucelus	L		27	-32	-36	12	5.5

L = left, R = right, Hemi = hemisphere; BA = Brodmann Area; k = cluster extent; x/y/z = MNI coordinates and T- values refer to maximally activated voxel for each significant cluster, voxel threshold $p < 0.05$ FWE-corrected, $k > 10$, anatomic structures have been determined using NITRC Mango (Kochunov et al., 2002)

Supplementary Table 3

b)

Naming/Reading Task (words block)

**Region**	**Hemi**	**Pre-treatment > Post-treatment**					
		**BA**	***k***	***x***	***y***	***z***	***T***
No significant cluster							

**Region**	**Hemi**	**Post-treatment > Pre-treatment**					
		**BA**	***k***	***x***	***y***	***z***	***T***
Middle frontal gyrus	L	11	17	-26	36	-14	6.1
		6	12	-54	8	40	5.8

L = left, R = right, Hemi = hemisphere; BA = Brodmann Area; k = cluster extent; x/y/z = MNI coordinates and T- values refer to maximally activated voxel for each significant cluster, voxel threshold $p < 0.05$ FWE-corrected, $k > 10$, anatomic structures have been determined using NITRC Mango (Kochunov et al., 2002)

Supplementary Table 4

a)

Naming/Reading Task (pictures block)

Region	Hemi	Pre-treatment > Post-treatment					
		BA	*k*	*x*	*y*	*z*	*T*
Culmen	L		23	-28	-34	-22	7.5
			19	-30	-54	-16	5.5
	R		10	12	-48	-22	5.5
Parahippocampus	L	36	23	-36	-32	-22	5.1
		30	45	-12	-46	8	6.1
Anterior cingulate	L	32	425	-14	48	-14	6
Inferior temporal gyrus	L	37	19	-58	-46	-22	5.6
Thalmus	L		80	-2	-26	-2	5.3
Middle frontal gyrus	L	10	425	-42	50	-12	6.3
		47		-46	38	-14	6.2
Inferior frontal gyrus	L	47	13	-52	36	-6	5.4
Inferior parietal lobule	R	40	15	68	-34	30	7.6
Postcentral parietal gyrus	L	40	15	-38	-30	62	6.9
**Region**	**Hemi**	**Post-treatment > Pre-treatment**					
		BA	*k*	*x*	*y*	*z*	*T*
Lateral global pallidus	R		13	30	-16	-10	6.3
Superior temporal gyrus	L	42	16	-72	-28	4	5.7
	L	22		-70	-20	2	5.6
Parahippocampus	L	36	19	-30	-18	-18	5.6
			10	-40	-22	-18	5.2
Superior frontal gyrus	R	10	38	26	52	-6	5.8
Middle frontal gyrus	L	10	17	-14	52	8	5.2

L = left, R = right, Hemi = hemisphere; BA = Brodmann Area; k = cluster extent; x/y/z = MNI coordinates and T- values refer to maximally activated voxel for each significant cluster, voxel threshold $p < 0.05$ FWE-corrected, $k > 10$, anatomic structures have been determined using NITRC Mango (Kochunov et al., 2002)

Supplementary Table 4

b)

Naming/Reading Task (words block)

Region	Hemi	Pre-treatment > Post-treatment					
		BA	*k*	*x*	*y*	*z*	*T*
No significant cluster							

Region	Hemi	Post-treatment > Pre-treatment					
		BA	*k*	*x*	*y*	*z*	*T*
Culmen	L		15	-6	-44	-18	6.1
	R		11	12	-40	-22	5.3
Fusiform gyrus	L	20	39	-44	-34	-16	7.1
Inferior temporal gyrus	L	37	39	-50	-42	-18	6.2
Middle frontal gyrus	R	10	10	12	64	-4	5.3

L = left, R = right, Hemi = hemisphere; BA = Brodmann Area; k = cluster extent; x/y/z = MNI coordinates and T- values refer to maximally activated voxel for each significant cluster, voxel threshold $p < 0.05$ FWE-corrected, $k > 10$, anatomic structures have been determined using NITRC Mango (Kochunov et al., 2002)
